# Insights into the molecular diversity of *Plasmodium vivax* merozoite surface protein-3γ (*pvmsp3γ*), a polymorphic member in the *msp3* multi-gene family

**DOI:** 10.1038/s41598-020-67222-z

**Published:** 2020-07-03

**Authors:** Napaporn Kuamsab, Chaturong Putaporntip, Urassaya Pattanawong, Somchai Jongwutiwes

**Affiliations:** 1 Molecular Biology of Malaria and Opportunistic Parasites Research Unit, Department of Parasitology, Faculty of Medicine, Chulalongkorn University, Bangkok, Thailand; 2 Inter-Department Program of Biomedical Sciences, Faculty of Graduate School, Chulalongkorn University, Bangkok, Thailand

**Keywords:** Microbiology, Parasitology

## Abstract

*Plasmodium vivax* merozoite surface protein 3 (PvMSP3) is encoded by a multi-gene family. Of these, PvMSP3α, PvMSP3β and PvMSP3γ, are considered to be vaccine targets. Despite comprehensive analyses of PvMSP3α and PvMSP3β, little is known about structural and sequence diversity in PvMSP3γ. Analysis of 118 complete *pvmsp3γ* sequences from diverse endemic areas of Thailand and 9 reported sequences has shown 86 distinct haplotypes. Based on variation in insert domains, *pvmsp3γ* can be classified into 3 types, i.e. Belem, Salvador I and NR520. Imperfect nucleotide repeats were found in six regions of the gene; none encoded tandem amino acid repeats. Predicted coiled-coil heptad repeats were abundant in the protein and displayed variation in length and location. Interspersed phase shifts occurred in the heptad arrays that may have an impact on protein structure. Polymorphism in *pvmsp3γ* seems to be generated by intragenic recombination and driven by natural selection. Most *P. vivax* isolates in Thailand exhibit population structure, suggesting limited gene flow across endemic areas. Phylogenetic analysis has suggested that insert domains could have been subsequently acquired during the evolution of *pvmsp3γ*. Sequence and structural diversity of PvMSP3γ may complicate vaccine design due to alteration in predicted immunogenic epitopes among variants.

## Introduction

Malaria caused by *Plasmodium vivax* is an important public health burden in tropical areas outside Africa. The presence of hypnozoites in *P. vivax*-infected individuals is responsible for chronic relapsing symptoms and compromise effective radical treatment^1^. Despite current control measures based on anti-malarials and insecticides, vaccine development is regarded as an adjunctive strategy to combat malaria^2^.

The merozoite surface coat of malaria parasites possesses several proteins implicated in recognition and invasion of host erythrocytes. The merozoite surface protein 3 (MSP3) is considered to be a vaccine candidate because anti-MSP3 antibodies elicited protective immunity in both mice and nonhuman primates as shown by a remarkable reduction in parasite density, inhibition of parasite growth and protection against parasite challenge^3,4^. The MSP3 proteins in *P. vivax* (PvMSP3) are encoded by a multi-gene family containing 12 gene members arranged in tandem^5^. Two of these members, *pvmsp3α* (PVX_097720) and *pvmsp3β* (PVX_097680), exhibit extensive sequence diversity among laboratory and field isolates^6–13^. Both PvMSP3α and PvMSP3β were immunogenic upon natural infections. Importantly, Papua New Guinean children who developed anti-PvMSP3α antibodies had a significant lower risk of symptomatic malaria^14^. Although the organizations of PvMSP3α and PvMSP3β are similar, characterized by conserved N- and C-terminal domains intervened by the alanine-rich coiled-coil central domain, sequences and the pattern of insertion/deletion polymorphism differ between these proteins. For example, a long stretch of deletion observed in the conserved C-terminal domain of PvMSP3β has not been found in PvMSP3α^7,10^. To date, little is known about sequence variation in the complete coding regions of other *pvmsp3* members^15^. Meanwhile, comparative analysis of the *pvmsp3* family has revealed that *pvmsp3γ* (PVX_097670) is the most abundantly expressed gene member during trophozoite development and elicits higher transcriptional level than *pvmsp3α* and *pvmsp3β* during schizont stage^5^. Taken together, it is likely that PvMSP3γ could confer some important roles in the life cycle of *P. vivax*.

To assess the genetic diversity and structural variation of *pvmsp3γ*, 118 complete coding sequences were analysed from isolates obtained from diverse endemic areas of Thailand. Results revealed spatial variation in genetic diversity while recurrent intragenic recombination and natural selection have contributed to structural and sequence diversity at this locus, an important issue for vaccine design.

## Results

### Size variation and *pvmsp3γ* haplotypes

Of 150*P. vivax* isolates from 5 provinces, 118 *pvmsp3γ* complete gene sequences were obtained after exclusion of multi-clonal infections. The distribution of samples in each endemic area is shown in Table 1. Yala and Narathiwat Provinces are located next to each other with similar malaria transmission; *P. vivax* isolates in these areas were considered herein to be the same population, referred to as Yala-Narathiwat population. Extensive size variation in *pvmsp3γ* was observed among Thai isolates, ranging from 1,755 to 2,925bp. In total, 77 haplotypes were identified whose sequences differed from those previously reported. Of these, 24, 30, 21 and 5 haplotypes were observed among isolates from Tak, Ubon Ratchathani, Chanthaburi Provinces and Yala-Narathiwat population. The low haplotype diversity (0.587) of *pvmsp3γ* in Yala-Narathiwat population indicated limited number of variants and a skew toward particular haplotypes. On the other hand, more evenly distributed haplotypes were observed in other endemic areas as shown by haplotype diversity ranging from 0.929 to 0.996. Meanwhile, the levels of nucleotide diversity (π) for *P. vivax* populations in Tak, Ubon Ratchathani and Chanthaburi Provinces were more than 1.5-fold greater than that for Yala-Narathiwat population (Table 1).

**Table 1:** The number of haplotypes, haplotype diversity and nucleotide diversity in *pvmsp3γ* by endemic area.

Province	n	No. nucleotide	M	S	H	*h* ± S.D.	π ± S.E.
Tak	31	1,755-2,925	225	198	24	0.981 ± 0.014	0.147 ± 0.005
Ubon Ratchathani	32	1,773-2,922	181	166	30	0.996 ± 0.009	0.138 ± 0.004#
Chanthaburi	31	1,791-2,925	155	142	21	0.929 ± 0.036	0.124 ± 0.004#
Yala & Narathiwat	24	1,842-2,895	131	127	5	0.587 ± 0.102	0.079 ± 0.003##
Total	118	1,755-2,925	252	217	77	0.968 ± 0.010	0.151 ± 0.005

n = number of isolates; M = the number of mutations; S = the number of segregating sites; h = haplotype diversity; π = nucleotide diversity. M and S are computed from all nonrepeat regions.

Tests of the hypothesis that π in Tak population equals the corresponding values in other populations: # *p* < 0.05; ## *p* < 0.001 (Z-tests).

### Domain organization of *pvmsp3γ*

**Figure 1:**
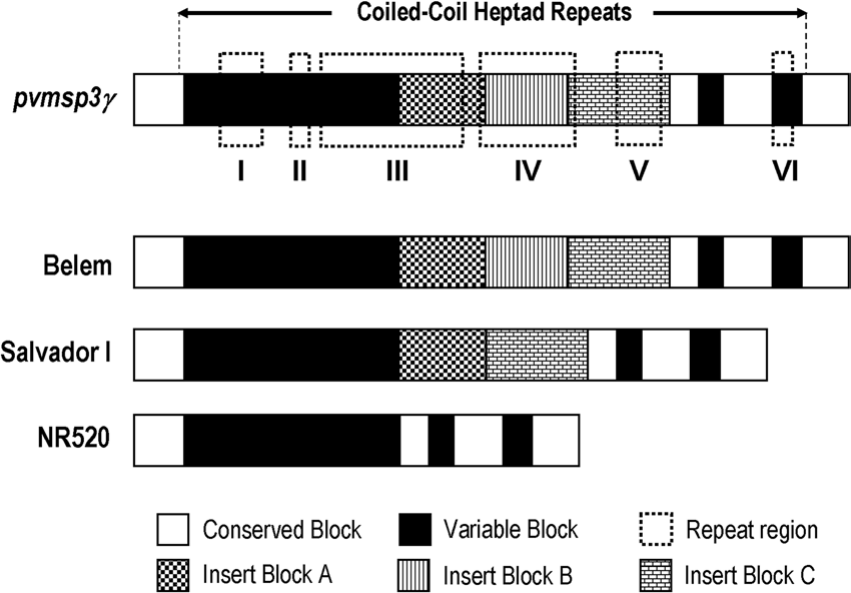
Domain organization of *pvmsp3γ*.

Analysis of 86 distinct *pvmsp3γ* haplotypes including Thai and 9 previously reported sequences has revealed that this locus can be partitioned into 10 domains based on the levels of nucleotide diversity and the presence/absence of long insertion/deletion (indel), comprising 4 conserved (π <0.1), 3 variable (π> 0.1) and 3 insert domains (Fig. 1, Table 2). The central domains contained 3 long stretches of indels, designated insert blocks A, B and C, corresponding to codons 347 to 450, 451 to 564 and 565 to 718, respectively, of the Belem sequence. Insert A was more polymorphic than other inserts (Table 2). Based on insert domains, *pvmsp3γ* can be divided into 3 groups, represented by the Belem, the Salvador I and the NR520 types. The Belem type, characterized by the presence of inserts A, B and C, was most common among Thai isolates (86 of 118 isolates, 72.9%)(Fig. 2). Five other published sequences including Brazil I, India VII, Indonesia I, North Korea I and Vietnam II, also belonged to the Belem type. The Salvador I type, lacking insert B, was found in 4 Thai isolates (3.4%), the Chesson and the Panama I strains. The Belem type contained 2,790 to 2,952 bp whereas the Salvador I type encompassed 2,537 to 2,562 bp. The newly identified NR520 type exhibited truncation of all inserts and displayed size variation from 1,755 to 1,863 bp. Although the NR520 type was found to circulate in all endemic areas in Thailand, it was predominantly detected among Yala-Narathiwat population (Fig. 2). Meanwhile, variable domain I was the most polymorphic as shown by the highest nucleotide diversity with 64 distinct haplotypes (Table 2).

**Table 2:** Nucleotide substitutions, the number of haplotypes (H), nucleotide diversity (*π*) and the rates of nucleotide substitutions at synonymous sites (*d*_S_) and nonsynonymous sites (*d*_N_) in *pvmsp3γ*.

Domain	No. nucleotide		n#	M§	S§	H	*π* ± S.E.	*d*_N_ ± S.E.	*d*_S_ ± S.E.
	Total	Repeats							
Conserved I	216	—	127	103	82	53	0.097 ± 0.012	0.067 ± 0.013	0.227 ± 0.041**
Conserved II	129	—	127	52	44	17	0.098 ± 0.015	0.120 ± 0.025	0.080 ± 0.032
Conserved III	216	—	127	56	50	31	0.070 ± 0.010	0.055 ± 0.011	0.083 ± 0.027
Conserved IV	204	—	127	46	45	30	0.065 ± 0.009	0.060 ± 0.010	0.068 ± 0.025
Variable I	726-834	426-513	127	221	207	64	0.220 ± 0.010	0.283 ± 0.021	0.242 ± 0.046
Variable II	99-108	—	127	75	52	21	0.178 ± 0.026	0.222 ± 0.036*	0.100 ± 0.033
Variable III	90-120	57-75	127	16	14	23	0.136 ± 0.023	0.151 ± 0.041	0.181 ± 0.108
Insert A	291-378	219-306	99	33	28	27	0.185 ± 0.015	0.103 ± 0.026	0.112 ± 0.042
Insert B	318-357	318-357	92	—	—	17	0.137 ± 0.012	—	—
Insert C	462	258	99	79	65	31	0.114 ± 0.009	0.103 ± 0.018	0.100 ± 0.022

#Thai isolates (n = 118) and worldwide strains (n = 9) including Belem (GenBank accession no. AF099663), Brazil I (AFMK01001074), North Korea I (AFNJ01000104), Salvador I (XM001613144), Indonesia I (KC907565), Chesson (KC907566), India VII (KC907567), Panama I (KC907568) and Vietnam II (KC907569). The numbers of isolates, haplotypes, mutations and segregating sites are represented by n, H, M and S, respectively. §Repeats are excluded. Tests of the hypothesis that mean *d*_S_ equals that for *d*_N_: *p < 0.05; **p < 0.0005 (Z test).

**Figure 2:**
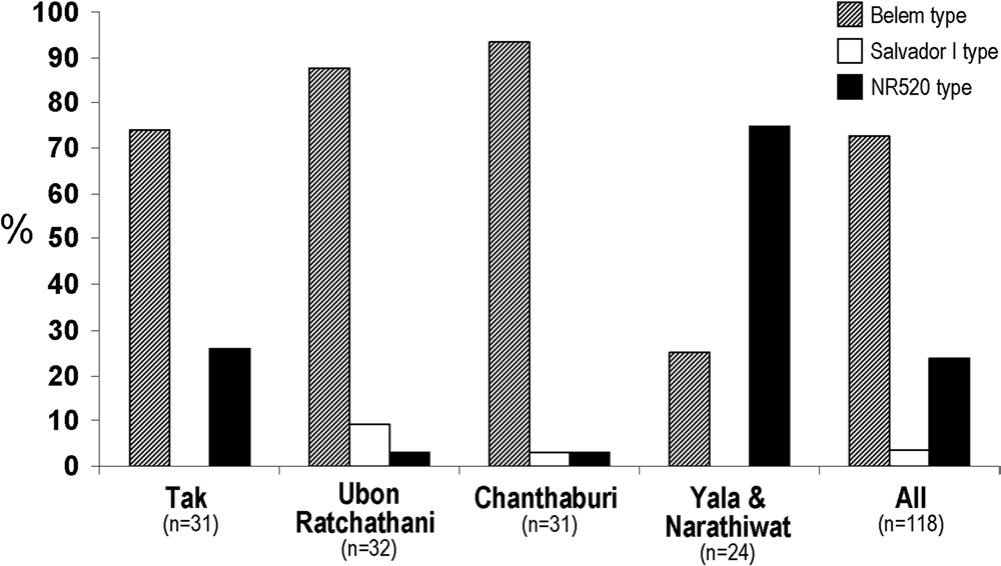
Distribution of major allelic types of *pvmsp3γ* in Thailand.

### Repeats in *pvmsp3γ*

Six regions in *pvmsp3γ* were found to possess imperfect or degenerate nucleotide repeats (Supplemental Table S1). These repeats did not uniformly occur at equivalent regions in each haplotype and were non-contiguous. Five of the repeat-containing regions encompassed the central coiled-coil structure of the protein (Fig. 1). No apparent tandem amino acid repeats was observed due to the imperfect nature of nucleotide repeats. The number of nucleotides in the consensus imperfect repeat motifs varied from 6 to 42 residues while the sequences were either related or unique. In total, 52 different repeat motifs have been identified (Supplemental Table S1). The most diverse repeat motifs were in repeat domain III, spanning the 5’ portion of variable domain I and the majority of insert block A (Fig. 1, Supplemental Table S1). The entire insert block B was composed of imperfect nucleotide repeats. Importantly, a number of repeat motifs in *pvmsp3γ* shared partial sequence similarity with those in the orthologous sequences in *P. cynomolgi* (*pcymsp3*), but not with *msp3* of *P. inui* (*pimsp3*) (Supplemental Table S2).

### Coiled-coil motifs and heptad breaks

The coiled-coil structure in a protein is formed by a heptad repeat pattern consisting of (*abcdefg*)_n_ where n is the number of repeat units. It is crucial that residues *a* and *d* are non-polar whereas *e* and *g* mostly possess oppositely charged residues^16^. Despite no tandem amino acid repeats, the central region of PvMSP3γ was mostly occupied by heptad repeats. The number of heptad repeat units in each haplotype were significantly correlated with the length of the proteins (Pearson’s *r* = 0.903, *p* = 1.60 × 10^−32^). The length of heptad repeats and their locations in the protein differed among haplotypes (Supplemental Fig. S1). Like other proteins containing coiled-coil structure, heptad breaks were found to be interspersed in the heptad repeat arrays. All types of phase shifts occurred in PvMSP3γ, characterized by insertion of 1 to 6 amino acids interrupting heptad arrays, with almost similar prevalence among the 3 types (Table 3). Insertion of 3 amino acid residues, known as stammer, was most common in both the Belem and the Salvador I types while insertion of 2 residues was predominantly observed among isolates bearing the NR520 type.

**Table 3:** Distribution of coiled-coil repeat motifs and heptad phase shift in PvMSP3γ.

Type	n*	% Heptad repeats in protein		% Phase shift in heptad repeats	Types of phase shift (%)					
		Range	Mean		+1	+2	+3	+4	+5	+6
Belem	72	38.4 - 57.8	50.7	12.3	1.1	0.7	4.1	2.1	2.1	2.2
Salvador I	6	41.0 - 44.7	42.5	10.0	0	1.9	3.9	2.3	1.9	0
NR520	8	35.6 - 44.3	40.1	12.0	2.2	4.4	2.9	1.5	0	1.1
Total	86	35.6 - 57.8	49.1	12.2	1.1	0.9	4.0	2.1	2.0	2.1

*Number of haplotypes. Types of phase shift are based on the number of amino acids (n < 7) that interrupt heptad repeat arrays. Phase shifts +1, +3 and +4 are skip, stammer and stutter, respectively. Sequences are from 118 Thai isolates and 9 sequences in the GenBank database as listed in the footnote of Table 2.

### Deviation from selective neutrality

To compare the rate of synonymous substitutions per synonymous site (*d*_S_) with that of nonsynonymous substitutions per nonsynonymous site (*d*_N_), regions containing repeats were excluded from analysis. A signature of purifying selection was observed in domain I of *pvmsp3*γ as shown by *d*_S_ significantly exceeding *d*_N_, implying functional or structural constraints in the N-terminus. By contrast, *d*_N_ significantly outnumbered *d*_S_ in variable domain II, suggesting positive selection. No deviation from selective neutrality was found in the remaining regions by domain-wise analysis (Table 2). Meanwhile, codon-based analyses excluding repeat regions were performed across 86 haplotypes by using the fast unconstrained Bayesian approximation (FUBAR)^17^. Of 125 substituted codons in conserved domains, FUBAR method detected 36 positively selected and 31 negatively selected codons. In variable domains, 40 of 103 substituted codons seem to have evolved under positive selection whereas four negatively selected codons were found. In insert domains, 11 positively selected codons and five negatively selected codons were identified (Supplemental Table S3).

### Recombination

By using the Recombination Detection Program^18^, 70 recombination breakpoints were identified in *pvmsp3γ* among Thai isolates with recombination distance varying from 49 to 2,782 bp (median 630 bp) (Table 4). There was 0.44 recombination site per 100 nucleotides in conserved domains which was comparable to that found in variable domains, i.e. 0.44-0.49 site per 100 nucleotides. By contrast, 0.26 recombination site per 100 nucleotides was observed in insert domains that was significantly less than that in conserved or in variable domains (*p* = 0.037 and 0.008, respectively). Meanwhile, almost comparable numbers of recombination breakpoints were detected for each *P. vivax* population from Tak, Ubon Ratchathani and Chanthaburi Provinces, containing 59, 54 and 53 recombination sites, respectively. By contrast, only 3 recombination breakpoints were found in Yala-Narathiwat population. Interestingly, the number of recombination breakpoints and the level of nucleotide diversity for each population exhibit a significant correlation (Pearson *r* = 0.972, *p* = 0.028).

**Table 4:** Distribution of recombination breakpoints in *pvmsp3γ* among Thai isolates.

Recombination	No. recombination breakpoints#	Distance (bp)	
		Range	Median
Within or between conserved domains	6	126-2782	1484
Within or between variable domains	16	49-2533	343
Within or between insert domains	9	106-437	246
Between conserved and variable domains	19	148-2758	808
Between conserved and insert domains	9	555-2279	1374
Between variable and insert domains	11	390-1350	791
Total	70	49-2782	630

*Consensus from 3 of 7 tests (RDP, GENCONV, Bootscan, MaxChi, Chimera, SiScan and TOPAL).

#Number counted from recombination events.

Recombination involved conserved domains = (34/765) x 100 = 0.44 site per 100 nucleotides.

Recombination involved variable domains = (46/939) x 100 to (46/1035) x 100 = 0.44-0.49 site per 100 nucleotides.

Recombination involved insert domains = (29/1110) x 100 = 0.26 site per 100 nucleotides.

### Population differentiation

The fixation indices (*F*_ST_) between Yala-Narathiwat population and populations elsewhere yielded significant values, ranging from 20.07% to 23.33% (*p* < 10^−5^). Despite the relatively low *F*_ST_ values, population differentiation occurred between parasite populations from Ubon Ratchathani and Chanthaburi Provinces, and between Tak and Chanthaburi Provinces, implying limited gene flow between these endemic areas. By contrast, gene flow seems to occur between populations from Tak and Ubon Ratchathani Provinces as shown by a non-significant *F*_ST_ value (Table 5).

**Table 5:** Genetic differentiation of *P. vivax* populations in Thailand based on the *pvmsp3γ* locus.

	Tak	Ubon Ratchathani	Chanthaburi	Yala & Narathiwat
Tak		0.0631	<10^−5^	<10^−5^
Ubon Ratchathani	0.0087		<10^−5^	<10^−5^
Chanthaburi	0.0390	0.0353		<10^−5^
Yala & Narathiwat	0.2093	0.2007	0.2333	

*F*_*ST*_ indices and their respective *p* values are in lower and upper diagonals, respectively.

### Gene tree

**Figure 3:**
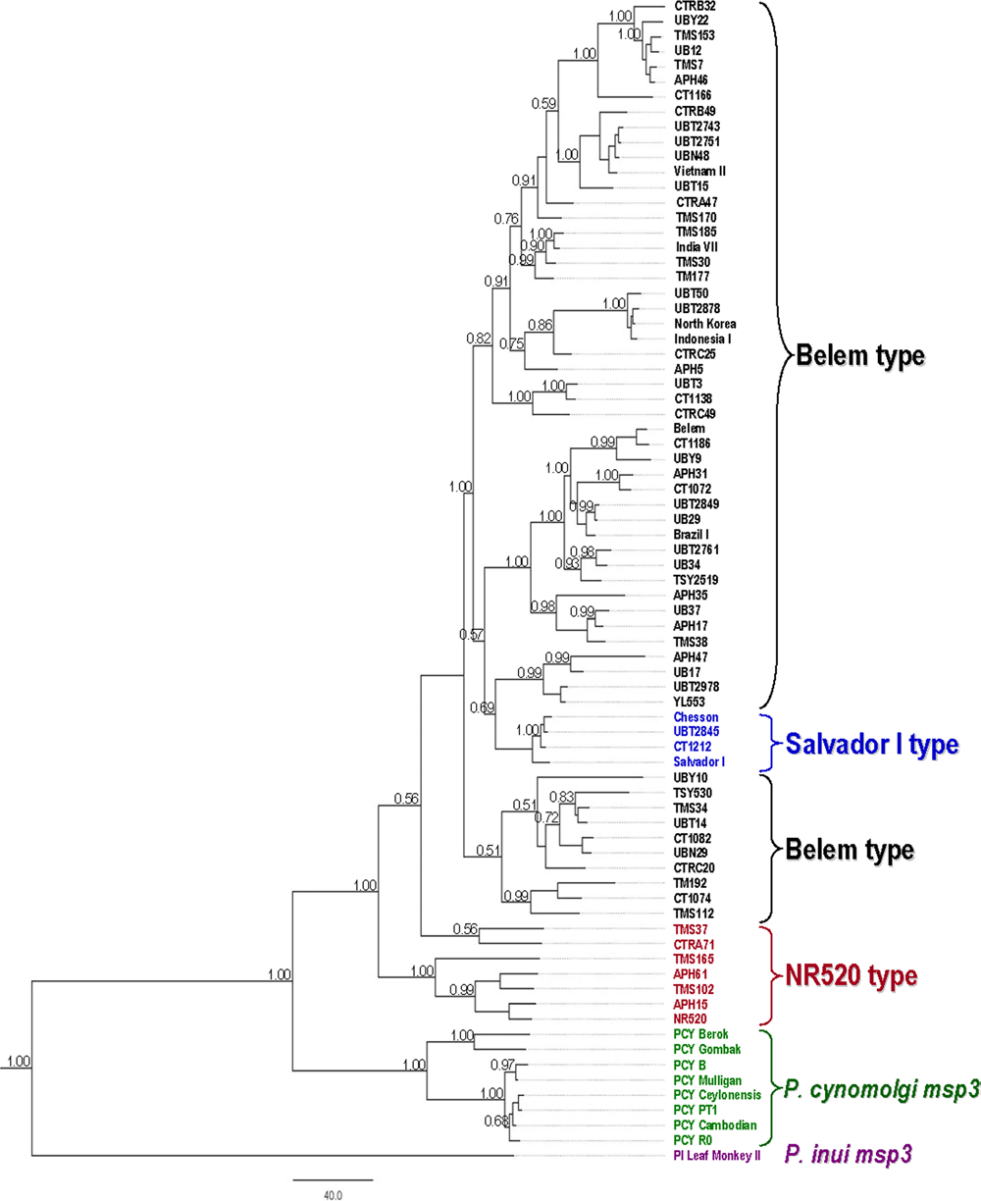
Bayesian tree of 77 representative distinct *pvmsp3γ* sequences inferred from concatenated conserved domains containing702 nucleotide sites in comparison with *pcymsp3* (GenBank accession nos. for PCY Cambodian, PCY PT1, PCY R0, PCY Ceylonensis, PCY Mulligan, PCY B, PCY Berok and PCY Gombak are KC907553-KC907559 and KC907561, respectively) and *pimsp3* (GenBank accession no. KC907508, Leaf Monkey II). Values on the branches indicate percentage of Bayesian posterior probability; only values greater than 0.50 are shown. Scale bar is the number of nucleotide substitutions per site. The *pvmsp3γ* sequence types are shown on the right with their orthologues.

Gene tree of *pvmsp3γ* was analysed by comparing with the currently known orthologues in *P. cynomolgi* (*pcymsp3*)(n = 8) and *P. inui* (*pimsp3*)(n = 1)^15^. The limited number of *pcymsp3* and *pimsp3* sequences has precluded analysis of structural organization of these genes; thereby, domain-wise sequence comparison was not possible. However, initial alignment of *pvmsp3γ* with *pcymsp3* and *pimsp3* has shown that comparable regions were confined to conserved domains of *pvmsp3γ*. Therefore, only conserved sequences in *pvmsp3γ* excluding repeats and indel sites were re-aligned with the *pcymsp3* and *pimsp3* sequences, yielding comparable 702 nucleotide positions. The gene tree inferred from the Bayesian Markov Chain Monte Carlo method yielded similar topology with those constructed by using the neighbour-joining and maximum likelihood methods in terms of relationship among *pvmsp3γ* types and genealogical relationship of orthologues (Fig. 3 and Supplemental Fig. S2). Although the evolutionary history of the resulting gene tree could have been affected by intragenic recombination and natural selection, all *pvmsp3γ* sequences were more closely related to *pcymsp3* than *pimsp3*. It is noteworthy that the Belem and the Salvador I sequences were nested within the NR520 type sequences. Meanwhile, all Salvador I sequences were clustered together and nested within the Belem sequences. Taken together, it is likely that the NR520 sequences could have evolved prior to the divergence of the Belem and the Salvador I types.

### Predicted linear B cell epitopes

By using the default cut-off value of 0.5 as suggested in the BepiPred 2.0 web-server for sequence-based epitope prediction^19^, the propensity for B cell epitopes seems to be abundant and encompasses almost the entire protein, sparring only short regions in both N- and C-terminal parts of all PvMSP3γ types (Supplemental Fig. S3). Therefore, truncation of insert blocks in the Salvador I and the NR520 types has decreased the number of linear B cell epitopes when compared with the Belem type.

### Predicted T helper epitopes

Based on some common HLA-DR haplotypes in Thai population, i.e. DRB1*0701, DRB*1202, DRB1*1501, DRB1*1502 and DRB1*1602^20^, *in silico* prediction of T helper epitopes in PvMSP3γ were analysed using the Belem sequence. The highest predicted scores for each of these HLA-DRB1 haplotypes have been mapped in different domains of the protein (Supplemental Table S4). For example, the highest predicted binding score for HLA-DRB1*0701 was found in the YLSGIPLLV peptide located at conserved domain I whose sequences contained 4 amino acid substitutions. One or more amino acid substitutions in this predicted epitope has resulted in approximately 25% reduction in HLA-DRB1*0701 binding scores. Meanwhile, amino acid substitutions in predicted T cell epitope (FAKIEAERA) for HLA-DRB1*1501 located at insert block A barely decreased the predicted binding scores while this predicted epitope was absence among 23.73% of Thai isolates due to deletion of this region (Supplemental Table S4). Furthermore, amino acid substitutions in predicted epitope (VAEAAKREI) in variable domain I have drastically lowered the binding score for more than 50%. Therefore, amino acid substitutions in predicted CD4 + T cell epitopes in PvMSP3γ may have immunological relevance.

## Discussion

The *pvmsp3γ* sequences displayed a higher level of nucleotide diversity than those observed in *pvmsp3α* and *pvmsp3β* of *P. vivax* populations in Thailand, i.e. 0.151, 0.033 and 0.088, respectively^10,15^. Although these 3 loci contained similar gene organization, the central coiled-coil domain in *pvmsp3α* has been partitioned into highly variable ‘block I’ and a relatively conserved ‘block II’^7^ while deletion of either the 5′ or the 3′ inserts, designated ‘insert A’ and ‘insert B’, respectively, occurred in *pvmsp3β*^10^. Deletion of one or more inserts in *pvmsp3γ* has suggested that this locus seems to be more reminiscent to *pvmsp3β* than *pvmsp3α* in the truncation pattern of the central coiled-coil domains. Likewise, a previous phylogenetic analysis has shown a closer relationship of *pvmsp3γ* with *pvmsp3β* than with *pvmsp3α*^15^.

Repeats are prevalent among proteins exposed to the surface of malaria parasites and have been suggested to preferentially induce T-independent B cell response that seems to be ineffective in eliminating infection^21,22^. However, no apparent tandem amino acid repeats was observed in PvMSP3γ although widespread imperfect nucleotide repeats have occupied the majority of the gene. Some nucleotide repeats in *pvmsp3γ* were located at regions containing coiled-coil heptad repeats predicted to form a bundle of intertwined α-helical structure. The occurrence of these predicted coiled-coil structures in the MSP3 family has suggested their structural or functional importance^23,24^. While the role of PvMSP3 remains elusive, proteins containing coiled-coil structure have been associated with diverse functions, such as skeletal proteins, proteins mediating oligomerisation and those providing protein-protein interaction sites for assembly and disassembly of protein complex^25,26^. Although a minimum of two heptads are reportedly required for a stable coiled-coil formation^25^, an overall more stabilized structure is formed by more heptad repeat units^26^. However, heptad breaks or phase shifts are frequently observed in proteins containing coiled-coil structure. Most phase shifts are reportedly confined to skip (an insertion of one amino acid), stutter (a deletion of 3 amino acids) and stammer (a deletion of 4 amino acids) while other types are less frequently encountered^16^. Although stammers are most common among heptad phase shifts in PvMSP3γ, discontinuities of heptad arrays other than stutters and skips are not uncommon among haplotypes. Phase shifts in heptad repeat arrays are responsible for alteration in coiled-coil structure of the protein. A stutter causes an increased pitch or unwinding of the supercoil structure whereas a stammer may cause overwinding of the helices^27^. A skip can disrupt the helical structure causing a local formation of a π turn while a break in the helical structure caused by either 2 or 6 amino acid insertions results in a local formation of a short β strand^28,29^. Therefore, variation in number of heptad repeat arrays, their distribution and differential occurrence of heptad breaks in PvMSP3γ may render structural variation of this protein among haplotypes.

Like several other merozoite surface proteins, PvMSP3α and PvMSP3β are immunogenic upon natural *P. vivax* infections^14,30–32^. Despite no seroepidemiological study on antibodies to PvMSP3γ, first identification of this gene was achieved by screening of the phage expression library with *P. vivax*-infected serum, suggesting that this protein could be immunogenic upon malaria exposure^24^. Although predicted linear B cell epitopes were abundantly found in PvMSP3γ (Supplemental Fig. S3), sequence heterogeneity in variable domains among major allelic types seems not to abolish the propensity of these predicted epitopes. Meanwhile, predicted linear B cell epitopes seem to encompass all insert domains in the Belem type sequences. Truncation of insert block B in the Salvador I type and the lack of all long inserts in the NR520 type have suggested variations in abundance of predicted linear B cell epitopes among PvMSP3γ variants. Intriguingly, antibody recognition of malarial surface protein may direct against conformational B cell epitopes. Importantly, approximately 75% of the coding region in PvMSP3γ has been predicted to contain coiled-coil structure (Fig. 1). It is therefore likely that structural variation in this protein exerted by variation in heptad repeat arrays, their distribution and the interruption by phase shifts in heptad repeats may alter the secondary or tertiary protein structure of PvMSP3γ. This in turn may confer some changes in conformational specificity of B cell epitopes if they would have been mapped in these regions.

Domain-wise analysis of *pvmsp3γ* has revealed evidence of purifying selection in conserved domain I that includes the N-terminal signal peptide. The predicted signal peptidase cleavage site between codons 25 (serine) and 26 (asparagine) in conserved domain I was perfectly conserved. Although substitutions at nearby residues V23I and E27K occurred in some isolates, they seem not to alter their likelihood of being signal peptidase cleavage site as predicted by SignalIP-5.0 web-server^33^. Domain-wise analysis has revealed evidence for positive selection in variable domain II of *pvmsp3γ* while codon-based analysis has detected 87 positively selected and 38 negatively selected codons in various domains (Supplemental Table S3). The majority of negatively selected codons occurred in conserved domains, particularly conserved domain I, consistent with results from domain-wise analysis. Likewise, positively selected codons were found more predominantly in variable domains. Meanwhile, most malarial surface proteins are subject to natural selection that could have been mediated by host immune pressure^34–38^. *In silico* analysis of PvMSP3γ peptides predicted to bind to common HLA-DR alleles among Thai population has suggested that the majority of amino acid substitutions in the predicted T helper epitopes differentially affect the peptide binding scores from slight to drastic changes (Supplemental Table S4). Because appropriate antibody production is mediated by T and B cell cooperation, failure to stimulate T helper cells that recognized processed antigens in association with HLA class II peptide may compromise effective immune responses.

Recombination is widespread in sexually reproducing organisms including malaria parasites. The observed significant correlation between the number of recombination sites in *pvmsp3γ* and the levels of nucleotide diversity among *P. vivax* populations in Thailand has implied that recombination is an important genetic mechanism conferring sequence diversity in this locus. It is noteworthy that *P. vivax* bearing the NR520 type predominated among Yala-Narathiwat population. Because several recombination breakpoints have been located at insert domains, deletions of all inserts in the NR520 type could be responsible for the lower number of recombination breakpoints and low level of genetic diversity in Yala-Narathiwat population (Table 4). Intriguingly, the absence of all inserts in the NR520 type along with its high prevalence could imply that these domains may be dispensable for parasite survival.

The widespread occurrence of imperfect repeats spanning almost half of the gene could have been generated by slipped strand mispairing or a related mechanism^39,40^. During DNA replication, strand slippage may occur, leading to misalignment of repetitive DNA regions. The consequences of this process could lead to an increase or decrease in these nucleotide repeat units. The diversity and complexity of repeat sequence motifs and the imperfect nature of the repeats in *pvmsp3γ* could have been generated by multiple rounds of replication slippage, or known as serial replication slippage, after several successive generations of the parasites and probably after a certain period of evolution^41^. It has been proposed that short tandem repeats can be expanded by slipped strand mispairing mechanism into longer tandem repeats while mutational changes can give rise to new sequence motifs. The longer the repeats are generated, the probability of noncontiguous slipped strand mispairing process increases by which the generation of long repeat motifs and the imperfect repeats may ensue^42,43^. Meanwhile, phylogenetic analysis has shown that the NR520 type sequences were placed basal to the Salvador I and the Belem types, suggesting that insert domains could have been subsequently acquired in the evolution of *pvmsp3γ* (Fig. 3). This finding seems to be in line with the notion that repeats arisen by chance can be expanded into longer repeats by slipped strand mispairing mechanism^39,42^. Meanwhile, the presence of similar and related repeat motifs observed in *pvmsp3γ* and *pcymsp3* has suggested that the generation of nucleotide repeats could have predated speciation of *P. vivax* and *P. cynomolgi*. On the other hand, the absence of sequence similarity of these repeat motifs in *pimsp3* has implied evolutionary divergence of *P. inui* lineage from *P. vivax* and *P. cynomolgi* lineages, consistent with closer genetic relatedness between *P. vivax* and *P. cynomolgi* than between *P. vivax* and *P. inui* based on analysis of the mitochondrial sequences^44,45^.

The number of haplotypes, nucleotide diversity and recombination sites in *pvmsp3γ* of Yala-Narathiwat population were remarkably lower than those in other endemic areas of Thailand, consistent with results from previous analyses of genes encoding thrombospondin-related adhesive protein (*pvtrap*), *pvmsp3β* and merozoite surface protein 7E (*pvmsp7e*)^10,36,37^. Although various factors could contribute to these findings, genetic bottleneck effects due to differential effectiveness in malaria control measures could play an important role^46^. It is noteworthy that trans-border migration of malaria patients was not uncommon in Tak, Ubon Ratchathani and Chanthaburi provinces whereas almost all malaria cases in Yala and Narathiwat provinces were indigenous and locally acquired. Although population structure has been observed in *P. vivax* populations from Chanthaburi, Yala and Narathiwat Provinces, evidence for gene flow between parasites from Tak and Ubon Ratchathani Provinces could have maintained and enhanced genetic diversity of *pvmsp3γ* in these areas.

In conclusion, *pvmsp3γ* exhibits extensive sequence diversity among Thai isolates. Sequence comparison has identified conserved, variable and insert domains in this locus with imperfect nucleotide repeats in variable and insert domains. Sequence diversity in *pvmsp3γ* could have arisen from intragenic recombination and natural selection while slipped strand mispairing mechanism elicited variation in sequence and length of the nucleotide repeats. Most *P. vivax* populations in Thailand exhibited population structure. Variation in the coiled-coil heptad repeats and amino acid substitutions in predicted immunogenic epitopes may affect host immune recognitions. Further studies are required to address whether sequence and structural diversity in PvMSP3γ would compromise vaccine development.

## Materials and Methods

### Study population

Approximately 2 mL of blood samples from 150 febrile malaria patients diagnosed by microscopy were obtained from Tak in 2005 and 2013 (n = 40), Ubon Ratchathani in 2009 and 2016 (n = 40), Chanthaburi in 2007 and 2011 (n = 40), Narathiwat in 2005 and 2009 (n = 15) and Yala Provinces during 2008-2009 (n = 15).

### DNA preparation, confirmation of *P. vivax* and genotyping

Genomic DNA was prepared by using QIAamp DNA mini kit (Qiagen, Hilden, Germany). Species-specific nested PCR was deployed for confirmation of *P. vivax* DNA per our previous report^47^. Clonal diversity was determined by allele-specific nested PCR targeting the polymorphic block 6 of the merozoite surface protein 1 of *P. vivax* (*PvMSP1*) as previously described (Supplemental Method 1)^37,48^.

### Amplification and sequencing *of pvmsp3γ*

The complete coding region of pvmsp3γ was amplified by PCR using primers pvmsp3γ-F (5′-TTTACTGCACAATTATGATCGC-3′) and pvmsp3γ-R (5′-CTCAAGTTATCCTAATTTGTGAAC-3′) based on GenBank accession no. AF099663. Amplification was performed in a total volume of 30 µL containing 0.2 µM of each primer, 200 µM dNTP, PCR buffer, nuclease free water, 2 µL of template DNA and 1.25 units of TaKaRa LA TaqTM (Takara, Seta, Japan). The amplification conditions consisted of a pre-amplification denaturation at 94 °C, 60 s; followed by 35 cycles of denaturation at 96 °C, 20 sec; annealing at 50 °C, 30 sec; and polymerization at 72 °C, 5 min, and a final elongation at 72 °C, 10 min using GeneAmp 9700 PCR thermal cycler (Applied Biosystems, Foster City, CA). PCR-amplified products were purified by QIAquick PCR purification kit (Qiagen, Hilden, Germany) and used as templates for bi-directional sequencing with ABI PRISM BigDye Terminator v3.1 Ready Reaction Cycle Sequencing kit (Applied Biosystems) and sequencing primers.

### Data analysis

The *pvmsp3γ* sequences were aligned by using the codon-based option in MUSCLE program with adjustment by eye^49^. Repetitive DNA sequence motifs were identified by using Tandem Repeats Finder version 4.0 program^50^. The coiled-coil heptad repeats were predicted by using the Paircoil 2 program^51^. Phase shifts or heptad breaks in the predicted helical domains were classified based on the number of amino acids that create discontinuities of the heptad repeats and classified as +1 to +6^16^. The number of haplotypes and haplotype diversity including its sampling variance were computed by using the DnaSP version 5.10 program^52^. Nucleotide diversity was calculated using maximum composite likelihood estimate implemented in the MEGA 6.0 program^53^. In non-repeat regions, *d*_*S*_ and *d*_*N*_ were calculated using Nei and Gojobori’s model with Jukes-Cantor correction and their standard errors by 1000 bootstrap pseudoreplicates^53^. Deviation from selective neutrality was also determined by codon-based approach with FUBAR method using the screened sequence data from the GARD program in the Datamonkey web-server in which the recombination segments were excluded^54^. Significance levels of codons deviated from neutrality were essentially followed the default values as suggested in the Datamonkey web-server^54^. Recombination Detection Program version 4 that includes RDP4, GENCONV, Bootscanning, the Maximum Chi Square, CHIMAERA, Sister Scanning and 3SEQ methods was deployed to detect evidences of intragenic recombination^18^. The *F*_ST_ value as a parameter for population differentiation was computed by using different hierarchical analyses of molecular variance implemented in the Arlequin software version 3.11 and the significance level by permutation test^55^. Phylogenetic trees were constructed by using (i) the Bayesian Evolutionary Analysis by Sampling Trees 2 (BEAST 2) package based on Markov Chain Monte Carlo algorithm^56^, (ii) the neighbour-joining tree based on maximum composite likelihood model implemented in the MEGA 6.0 program and (iii) the maximum likelihood tree based on the Tamura-Nei parameter with discrete Gamma distribution to model evolutionary rate and some evolutionarily invariable sites inferred from the best model for the sequence data. The Bayesian tree was constructed by using uncorrelated lognormal relaxed clock, Tamura-Nei substitution model, coalescent constant population and a 4 category gamma site heterogeneity model. Simulations were run for 10,000,000 cycles and logged at every 1,000 cycles. Confidence levels of tree branching patterns for the Bayesian tree were retrieved from the percentage of posterior probability implemented in the DensiTree program of the BEAST 2 package^56^. Reliability of the trees inferred from the neighbour-joining and maximum likelihood methods was assessed by 1,000 bootstrap pseudoreplicates using the MEGA 6.0 program^53^. Linear B cell epitopes were predicted based on a random forest algorithm trained on epitopes annotated from antibody-antigen protein structures implemented in BepiPred 2.0 web-server^19^. CD4 + T-cell epitopes were predicted using the The Immune Epitope Database (IEDB) webserver^57^ by taking into account some HLA-DR alleles common among Thai population^20^.

### Accession numbers

Seventy-seven complete coding sequences of *pvmsp3γ* have been deposited in NCBI GenBank under accession numbers MT363114-MT363190.

### Ethical Approval

This study was reviewed and approved by the Institutional Review Board in Human Research of Faculty of Medicine, Chulalongkorn University, Thailand (IRB No. 546/58 and COA No. 041/2016). Prior to blood sample collection, informed consent was obtained from all participants or from their parents or guardians. All procedures were performed in accordance to the relevant guidelines and regulations.

## Supplementary information

Supplementary Information.

## Data Availability

The datasets generated during and/or analyses during the current study are available from the corresponding author upon request.
