# Supplementary material for: Insights into the molecular diversity of Plasmodium vivax merozoite surface protein-3γ (pvmsp3γ), a polymorphic member in the msp3 multi-gene family
Source: Sci Rep. 2020 Jul 3;10:10977. doi: 10.1038/s41598-020-67222-z (PMC7335089; doi:10.1038/s41598-020-67222-z)
Supplement: Supplementary file 1 — Supplementary Information. [file 41598_2020_67222_MOESM1_ESM.pdf]

Insights into the molecular diversity of *Plasmodium vivax* merozoite surface protein-3γ (*pvmsp3γ*), a polymorphic member in the *msp3* multi-gene family

Napaporn Kuamsab[1,2], Chaturong Putaporntip[1,*], Urassaya Pattanawong[1], Somchai Jongwutiwes[1]

[1]Molecular Biology of Malaria and Opportunistic Parasites Research Unit, Department of Parasitology, Faculty of Medicine, Chulalongkorn University, Bangkok, Thailand.

[2]Inter-Department Program of Biomedical Sciences, Faculty of Graduate School, Chulalongkorn University, Bangkok, Thailand

SUPPLEMENTARY INFORMATION

Contents

Method S1. Genotyping of *pvmsp1* based on polymorphic block 6…………………….……....2

Table S1. Consensus nucleotide repeat motifs in *pvmsp3γ*…………………………..…….…4

Table S2. Common consensus repeats in *pcymsp3* and *pvmsp3γ* shown in blue residues…...…5

Table S3. Distribution of codons deviated from selective neutrality in *pvmsp3γ* …………….....6

Table S4. Predicted HLA-DR bound peptides in PvMSP3γ of 118 Thai isolates……………….7

Figure S1. Coiled-coil heptad repeats in PvMSP3γ haplotypes…………………………………9

Figure S2. Neighbour-joining and maximum likelihood trees inferred from conserved domains of *pvmsp3γ* and orthologous regions in *pcymsp3* and *pimsp3*….…………..12

Figure S3. Plots of predicted scores for linear B-cell epitopes across PvMSP3γ………………13

Method S1. Genotyping of *Plasmodium vivax* based on blocks 5, 6 and 8 of the merozoite surface protein 1 (*pvmsp1*) gene

Background: The polymorphic single-copy *pvmsp1* gene encodes a GPI-anchored merozoite surface protein with a molecular weight of ~200 kDa. The *pvmsp1* locus can be divided into 13 blocks consisting of 6 variable and 7 conserved blocks (1)(Fig). Block 6 can be partitioned into 5' and 3' portions bearing either the Salvador I or the Belem sequence types. A recombination site has been observed in the middle portion of block 6 among field isolates, resulting in four different possible combination, characterized by (i) 5'-Salvador I/Salvador I-3', (ii) 5'-Belem/Belem-3', (iii) 5'-Salvador I/Belem-3' and (iv) 5'-Belem/Salvador I-3'. The 3' portion of the Belem type contains sequence encoding variable numbers of polyglutamine repeats. Therefore, size variation can be observed among alleles bearing the Belem sequence type at the 3' portion of block 6. Although block 5 has been assigned as conserved block, a small cluster of dimorphic nucleotide substitutions occurs near the 5' portion of this block. Meanwhile, variable block 8 possesses highly divergence dimorphic alleles. Evidence of interallelic recombination between blocks 5 and 8 has been reported among field isolates (1-3).

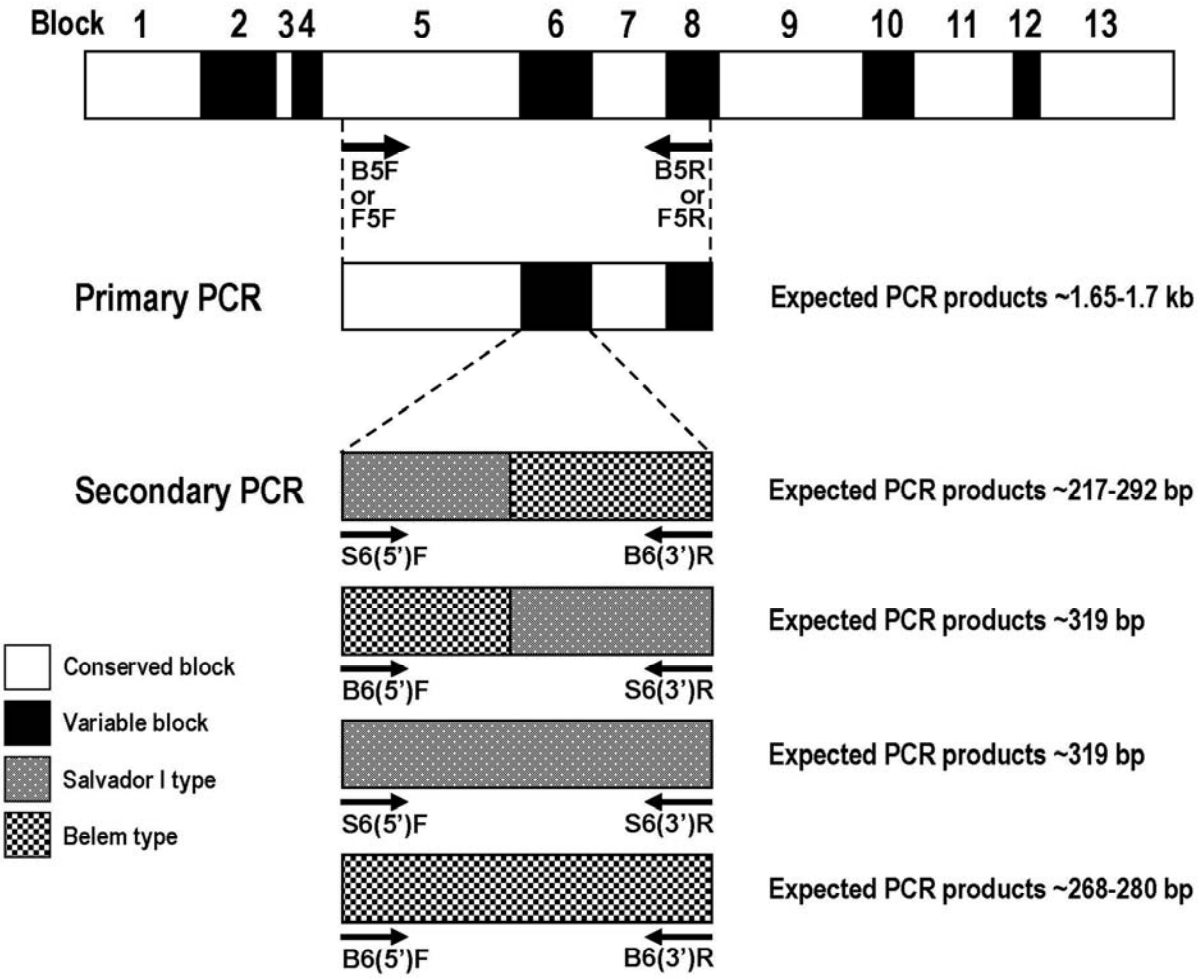

Figure 1. Scheme of *pvmsp1* depicting primer locations for genotyping.

Genotyping strategy:
Step 1: Genotyping of blocks 5 and 8

DNA fragments encompassing blocks 5 and 8 were amplified by PCR using a pair of four possible combinations of allele-specific primers in separate PCR reactions. The forward primers were B5F (5'-GCACCAAATCAGCAG -3', after the Belem sequence, GenBank accession no. AF435594) and F5F (5'-AAGTGCATCGAGTACCA -3', Thai-F127 sequence, GenBank accession no. AF435607), and the reverse primers B8R (5'-AACCACAACAACAGTTACT-3', Belem sequence) and E8R (5'-GTTGCACCTAATTCAGGCAA -3', Thai E26 sequence, GenBank accession no. AF435605)(Fig). The PCR profile contained 35 cycles of 92°C for 30 sec, 60°C for 30 sec and 72°C for 1.30 min, respectively, followed by a final primer extension at 72°C for 5 min in a thermal cycler (GeneAmpR PCR System 9700, Applied Biosystems, USA). Amplification was done in 25 µl reaction mixture containing *P. vivax* DNA, 200 µM each dNTP, 2.5 µl of 10x Taq buffer, 0.4 units of EXTaq DNA Polymerase (Takara, Japan) and 0.25 µM of primers. Analysis of PCR products was performed by using 1.5% agarose gel electrophoresis. If the resulting PCR yielded a positive band spanning ~1.65-1.7 kb from two or more PCR reactions, it is concluded to be multiclonal infection of *P. vivax*. When positive results were obtained from only one of these four different combinations of PCR primers, further genotyping of block 6 was performed.

Step 2: Genotyping of block 6

The amplified PCR products generated from step 1 were used as templates to determine alleles of blocks 6(5') and 6(3') in 4 separate amplification reactions using block 6-specific primers. Primer sequences were B6(5')F: 5'-CCAAGCCGGCAGCGTCAGCGCCAGTGACCA-3', B6(3')R: 5'-GTATCAGCCACACCAGCA-3', S6(5')F: 5'-AGATATAGTGGCAAAGGGA CAA-3' and S6(3')R: 5'-CCCACCATCGACACCT-3' in which B6(5')F and B6(3')R were derived from the Belem sequence and S6(5')F and S6(3')R from the Salvador I sequence (GenBank accession no. AF435593). Four separate PCR amplifications were performed using four different combinations of primers, i.e. (i) B6(5')F and B6(3')R, (ii) S6(5')S and S6(5')R, (iii) B6(5')F, and S6(3')R and S6(5')F and B6(3')R. PCR were performed in separate The secondary PCR contained 25 µl reaction mixtures and the thermal cycler profile was the same as for the primary PCR, except using 25 cycles of amplification and polymerization for 1 min. Secondary PCR products were separated by electrophoresis in 2.8% agarose gel electrophoresis and stained with eithidium bromide and visualized under UV transillumination. The resulting PCR products were 217 to 319 bp, depending on the allelic types of block 6 (Fig). A single-band PCR product generated from only one of four different combinations of primers was classified as single clone infection of *P. vivax* based on genotyping of block 6 of *pvmsp1*.

References

1. Putaporntip, C. et al. Mosaic organization and heterogeneity in frequency of allelic recombination of the *Plasmodium vivax* merozoite surface protein-1 locus. *Proc. Natl. Acad. Sci. U.S.A.* **99**, 16348-1653. (2002).
2. Putaporntip, C., Jongwutiwes, S., Seethamchai, S., Kanbara, H. & Tanabe, K. Intragenic recombination in the 3' portion of the merozoite surface protein 1 gene of *Plasmodium vivax. Mol. Biochem. Parasitol.* **109**, 111-119 (2000).
3. Putaporntip, C., Jongwutiwes, S., Tanabe, K. & Thaithong, S. Interallelic recombination in the merozoite surface protein 1 (MSP-1) gene of *Plasmodium vivax* from Thai isolates. *Mol. Biochem. Parasitol.* **84**, 49-56 (1997).

Table S1. Consensus nucleotide repeat motifs in *pvmsp3γ*

Repeat Domain*	Motif	Sequence (5′-3′)
**I**	1	AAAGCAAAAGAAGTAGCAAAT
	2	AAAAAAAGAAGAAGCAGC
	3	AAAAAAATAGAAGAAGCAGAA
	4	AAAGTAAAAGAAGCAGAAGAA
	5	AAAAAAGCAAAGAAGCAGCA
	6	AAAAAAGCAAAAGAAACAGC
	7	AAAAAATAGTAGAAGCAGC
	8	AAAGCAAAAGAAGCAGCAAGAA
	9	AAAGAATGCAGCAAAAAATGCA
	10	AAAAAATGCAAAGAAAAGCAGC
	11	AAAGCAAAAAAAGCACCAAAA
	12	AAAAAAAGCAAAAAAAACAGCAAAGGACAAAGC
	13	AAAAAAAGCAGCAGAAAAAGC
**II**	1	AAGCAAAAAAAGAAGCAGAGA
	2	AAGGCGAAAAAAGAAGCAGAT
	3	AAGCACAAAAAGAAGCAGAAA
**III**	1	CAACTAAAGCATCAACTGAAG
	2	CAACTGAAGCAACTAAAGCAT
	3	CAACTAAAGCAACAAATGCAT
	4	CAACTGAAGCAACAAAACCAT
	5	CAACAAAAGCATCAAATAAAG
	6	CAACTGAAGCAACAAATGCAT
	7	CAACTAAACAACAAATGAAG
	8	CAACAGAAGCATCAAATAAAG
	9	CAACTAAAGCAACGGAAGCAT
	10	CAACAGAAGCATCAACTAAAG
	11	CAACAGAAGCAGCAACAAAAG
	12	CAAACAAAAGCATCAAATGAAG
	13	CAAATAAAGCAAAAGAAGCAT
	14	CAACTAAAGCAAAAGAGGCAT
	15	CAACTAAAGCAAAAGTAGCAT
	16	CAACTAAAGCAAAGAAGCAT
	17	CAACTAAGGCAAAGAAGCAT
	18	CAACTAAAGCAGCACAAGCAT
	19	CAAATAAAGCAT
	20	CAACTAAAGGCAT
	22	AATCGGCAACCGAAGCAGCAC
	23	AAACAGCAGCAGAAGCAGCAA
	24	GAAGCATCAAGAAAGGAAGCAAATGTAAAAGAAAAAACAGAC
**IV**	1	GCAGAA
	2	GCAGCAAAAGCAGAA
	3	GCAAAACAAGAAGCAAAAAAA
**V**	1	GCAAAAGCAGCAGCTGAAAATGCACAAAG
	2	GCAAAAACAGCAGCAGAA
	3	GCAAAAGCAGCA
	4	GCAGCAGAA
	5	GCAGCAAAAACA
**VI**	1	GAAGAAGAT
	2	GAAAGGAAGAT
	3	GAAGGAGACG
	4	AAGCAGCAGAATCTGCATCGA

*Domains are after Figure 1.

Table S2. Common consensus repeats in *pcymsp3* and *pvmsp3γ* shown in blue residues.

pcymsp3		*pvmsp3γ*		
GenBank Accession	Consensus Sequence	Repeat Domain	Motif	Consensus Sequence
KC907558	AAGCACAAAAAGAAGCAGAGA	II	1	AAGCAAAAAAAGAAGCAGAGA
	CGAAGCAGCACAATCTGCAAC	VI	4	AAGCAGCAGAATCTGCATCGA
	AACAGAAGCAGAAAAAGCACAA	III	11	CAACAGAAGCAGCAACAAAAG
	GAAGAAGGAGAC	I	3	AAAAAAATAGAAGAAGCAGAA
KC907559	CCGAAGCAGCACAATCTACAA	VI	4	AAGCAGCAGAATCTGCATCGA
	AACAGAAGCAGAAAAAGCACAA	III	11	CAACAGAAGCAGCAACAAAAG
KC907561	GCAACCGAAGAAGCACAATCT	I	3	AAAAAAATAGAAGAAGCAGAA
	AAACGAAGAACCACAATCTGCAACCGAAGCAGCAAAATCTG	III	22	AATCGGCAACCGAAGCAGCAC
KC907556	AAGCACAAAAAGAAGCAGAGA	II	1	AAGCAAAAAAAGAAGCAGAGA
	CGAAGCAGCACAATCTGCAAC	VI	4	AAGCAGCAGAATCTGCATCGA
	AACAGAAGCAGAAAAAGCACAA	III	11	CAACAGAAGCAGCAACAAAAG
	GAAGAAGGAGAC	I	3	AAAAAAATAGAAGAAGCAGAA
KC907554	AAGCACAAAAAGAAGCAGAGA	II	1	AAGCAAAAAAAGAAGCAGAGA
	AAGCACAAAAAGAAGCAGAGA	II	1	AAGCAAAAAAAGAAGCAGAGA
	AACAGAAGCAGAAAAAGCACAA	III	11	CAACAGAAGCAGCAACAAAAG
	GAAGAAGGAGAC	I	3	AAAAAAATAGAAGAAGCAGAA
KC907553	AAGCACAAAAAGAAGCAGAGA	II	1	AAGCAAAAAAAGAAGCAGAGA
	CGAAGCAGCACAATCTGCAAC	VI	4	AAGCAGCAGAATCTGCATCGA
	AACAGAAGCAGAAAAAGCACAA	III	11	CAACAGAAGCAGCAACAAAAG
	GAAGAAGGAGAC	I	3	AAAAAAATAGAAGAAGCAGAA

* Repeats in KC907553 are identical with those in KC907555 and KC907557. Repeat domains are after Figure 1 and Supplemental Table S1.

Table S3. Distribution of codons deviated from selective neutrality in *pvmsp3γ* of 118 Thai isolates determined by fast unconstrained Bayesian approximation (FUBAR) method

Domain§	Total codons	No. substituted codons	No. positively selected codons	No. negatively selected codons
Conserved I	72	39	4	15
Conserved II	43	24	11	3
Conserved III	72	30	11	7
Conserved IV	68	32	10	6
**Total**	**255**	**125**	**36**	**31**
Variable I	87	69	24	2
Variable II	35	26	14	2
Variable III	10	8	2	0
**Total**	**132**	**103**	**40**	**4**
Insert A	24	15	4	2
Insert C	68	35	7	3
**Total**	**92**	**50**	**11**	**5**

§Repeat regions and indels are excluded. Analysis was performed by using screened data excluding recombination segments from GARD analysis in the Datamonkey web-server [54].

Table S4. Predicted HLA-DR bound peptides in PvMSP3γ of 118 Thai isolates

HLA-DR	Predicted epitope*	Domain	Score	Haplotype#	Prevalence (%)
**B1*0701**	**YLSGIPLLV**	**Conserved I**	**84.70**	**Belem**	**2.54**
	HFAGIPLLV	Conserved I	64.22	TMS153	50.85
	HLSGIPLLV	Conserved I	64.22	CTRB32	30.51
	HFSGIPLLV	Conserved I	64.22	UBT2761	6.78
	HFPGIPLLV	Conserved I	64.22	UB17	0.85
	HFAGIHLLV	Conserved I	62.09	UBT3	5.08
	QFCGIPLLV	Conserved I	60.90	APH31	3.39
**B1*1202**	**VAEAAKREI**	**Variable I**	**87.97**	**Belem**	**21.19**
	VAESAKREI	Variable I	75.00	APH47	4.24
	EAENANGKI	Variable I	72.47	UBT2751	0.85
	VAEDANRGI	Variable I	71.60	TMS165	0.85
	EAEAAKREI	Variable I	70.42	TMS37	1.69
	LAEAANKKI	Variable I	65.46	APH15	17.80
	EAEAAKGEI	Variable I	63.77	APH35	0.85
	EAEAANREI	Variable I	60.54	UBT3	5.93
	EAEAANMEI	Variable I	58.01	UB17	2.54
	EAEAAKTEI	Variable I	57.42	UBT101	3.39
	EAEAANEEI	Variable I	51.13	TSY530	20.34
	EAESAKGEI	Variable I	50.14	APH5	10.17
	EAEDANGKI	Variable I	46.73	UBT2743	1.69
	EAEKAQKEI	Variable I	42.12	TM192	8.47
**B1*1501**	**FAKIEAERA**	**Insert A**	**88.00**	**Belem**	**26.27**
	FAKIGAERA	Insert A	88.00	UB23	5.93
	LAKIEAERA	Insert A	86.57	TMS38	12.71
	LAKIEAERA	Insert A	86.57	APH31	9.32
	LAKIEAESA	Insert A	86.57	TMS185	7.63
	LAKIEAEGA	Insert A	86.57	UBT2743	2.54
	LAKIEAERA	Insert A	86.57	APH31	2.54
	LAKIEAERA	Insert A	86.57	TM192	1.7
	FAKIETERA	Insert A	84.85	UBT2761	3.39

HLA-DR	Predicted epitope*	Domain	Score	Haplotype#	Prevalence (%)
	LAKVEAERA	Insert A	82.57	TSY530	4.24
	deletion	No Insert A	-	NR520	23.73
**B1*1502**	**FHALIAEKA**	**Variable I**	**86.28**	**Belem**	**22.88**
	FHAFLAEKA	Variable I	92.53	TMS112	9.32
	FHSFIAVKA	Variable I	90.99	TSY530	1.7
	FYTIIAEKA	Variable I	88.41	APH31	2.54
	FHAIVAERA	Variable I	88.41	TMS37	1.69
	FHAIIAEKA	Variable I	88.41	APH35	0.85
	FHAIVAEMA	Variable I	88.41	TMS185	0.85
	FHVLVAETA	Variable I	86.28	TMS165	0.85
	FHAVIAEKA	Variable I	84.67	UBT2743	2.54
	FHAFLAEKE	Variable I	82.44	TMS34	9.32
	FHAGNAVTA	Variable I	78.48	APH15	17.8
	FHAIVAERE	Variable I	78.32	TM192	8.47
	FHAGDAITA	Variable I	77.36	APH26	3.39
	ILAHIAEKA	Variable I	75.14	UBT3	12.71
	LYAGNAVTA	Variable I	71.43	UBT101	3.39
	ILVQIAEKA	Variable I	69.94	UB17	1.7
**B1*1602**	**DDDFKNLQT**	**Conserved IV**	**80.56**	**Belem**	**23.73**
	GDDFKNLQT	Conserved IV	78.42	TMS37	22.88
	DDDFKNLQK	Conserved IV	71.25	UBY22	53.39

* The highest scores for the Belem sequence are in bold. Amino acid substitutions are underlined.
# Representative haplotypes.

Figure S1. Coiled-coil heptad repeats in PvMSP3γ haplotypes

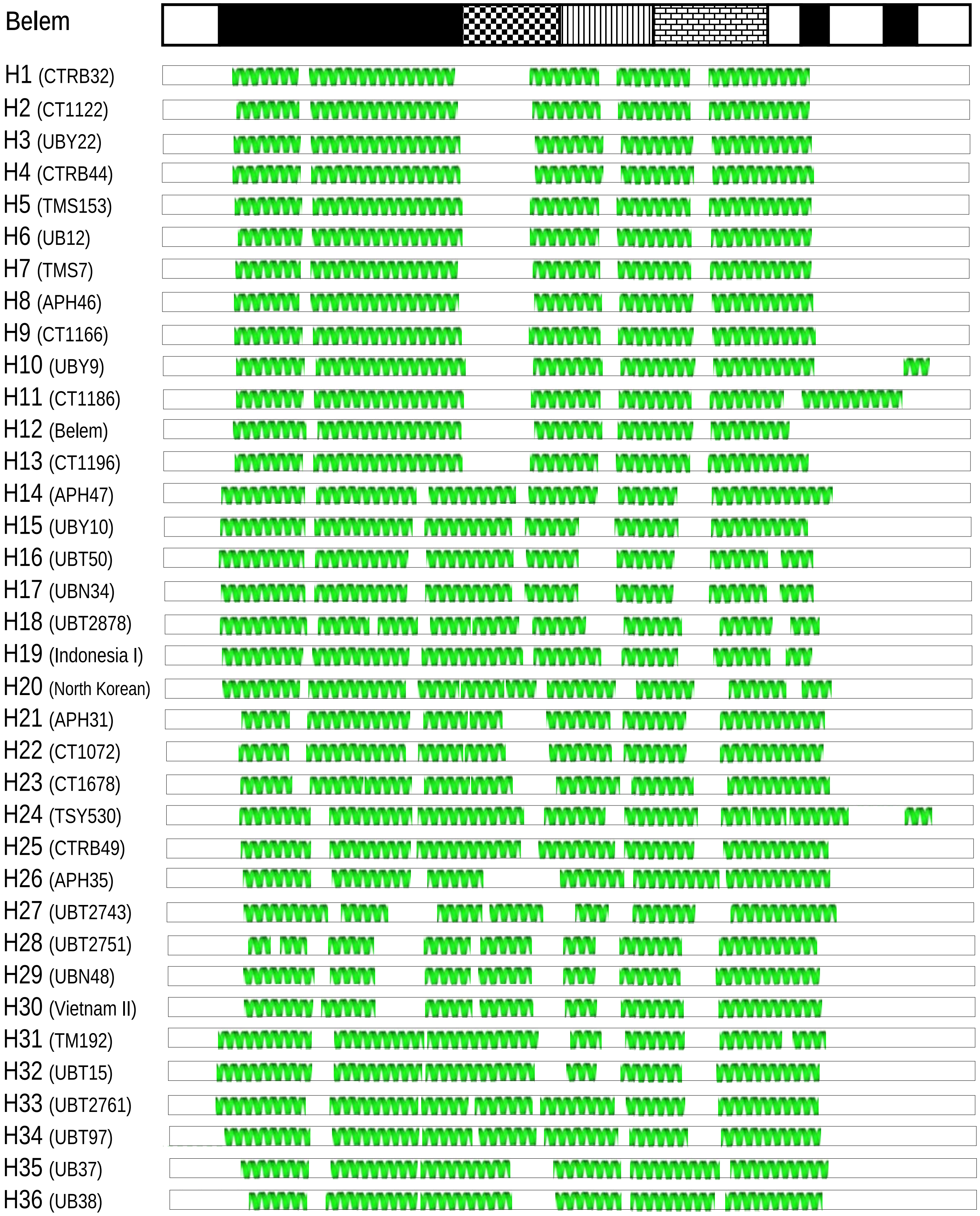

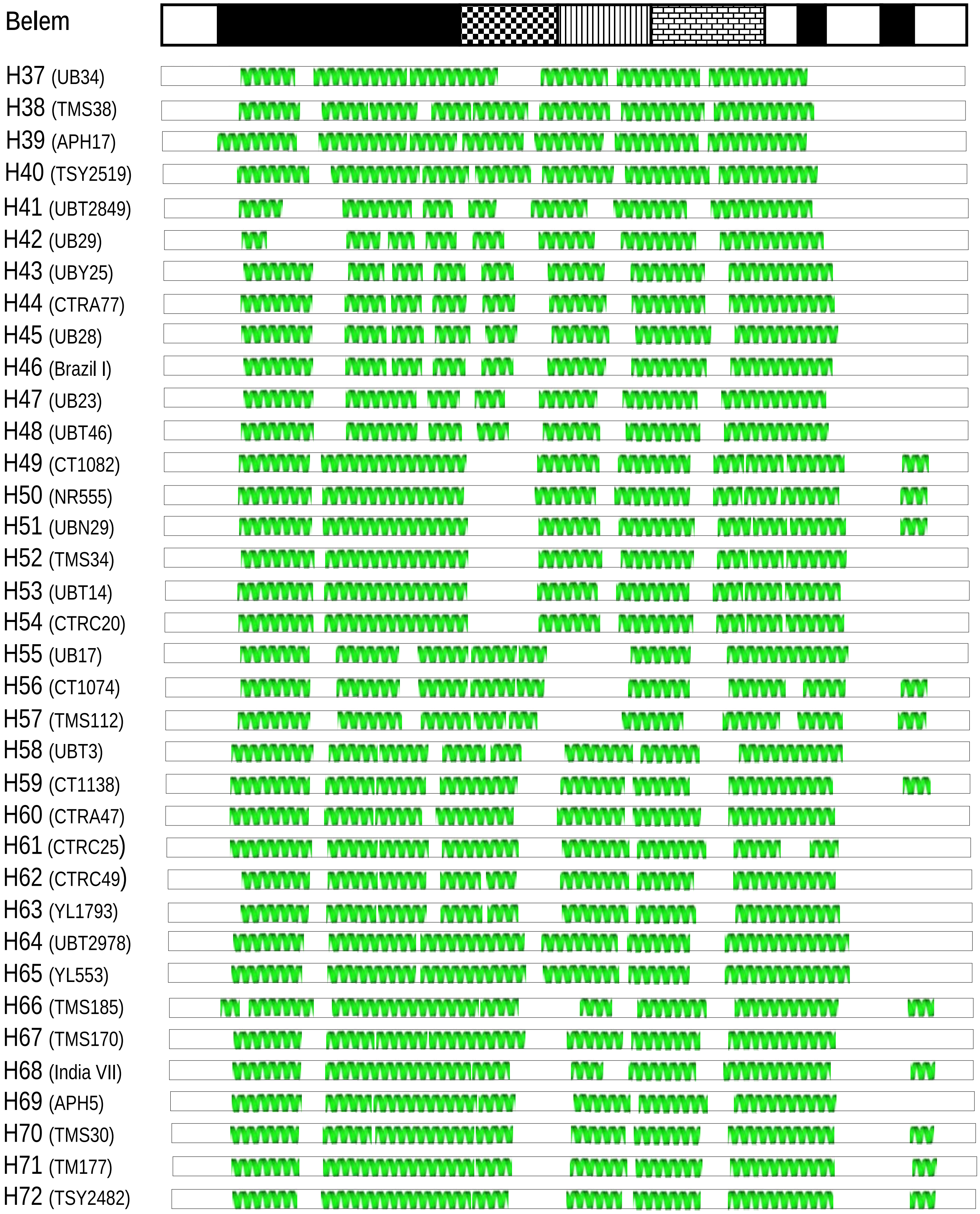
Belem
H37 (UB34)
H38 (TMS38)
H39 (APH17)
H40 (TSY2519)
H41 (UBT2849)
H42 (UB29)
H43 (UBY25)
H44 (CTRA77)
H45 (UB28)
H46 (Brazil I)
H47 (UB23)
H48 (UBT46)
H49 (CT1082)
H50 (NR555)
H51 (UBN29)
H52 (TMS34)
H53 (UBT14)
H54 (CTRC20)
H55 (UB17)
H56 (CT1074)
H57 (TMS112)
H58 (UBT3)
H59 (CT1138)
H60 (CTRA47)
H61 (CTRC25)
H62 (CTRC49)
H63 (YL1793)
H64 (UBT2978)
H65 (YL553)
H66 (TMS185)
H67 (TMS170)
H68 (India VII)
H69 (APH5)
H70 (TMS30)
H71 (TM177)
H72 (TSY2482)

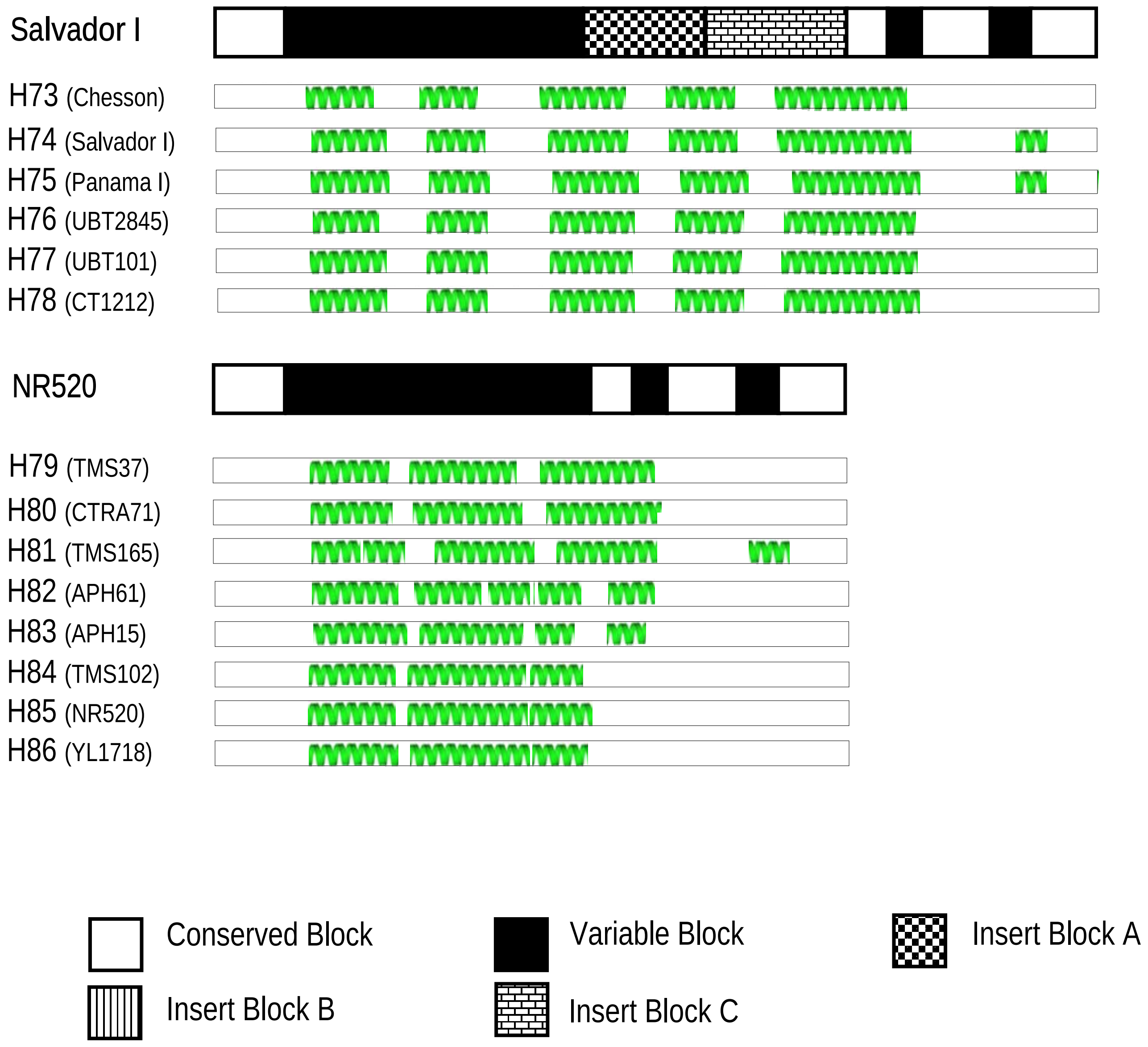
Salvador I
H73 (Chesson)
H74 (Salvador I)
H75 (Panama I)
H76 (UBT2845)
H77 (UBT101)
H78 (CT1212)
NR520
H79 (TMS37)
H80 (CTRA71)
H81 (TMS165)
H82 (APH61)
H83 (APH15)
H84 (TMS102)
H85 (NR520)
H86 (YL1718)
Conserved Block
Variable Block
Insert Block A
Insert Block B
Insert Block C

Figure S2. Neigbour-joining (A) and maximumlikelihood (B) trees inferred from conserved domains of *pvmsp3γ*, *pcymsp3* and *pimsp3*

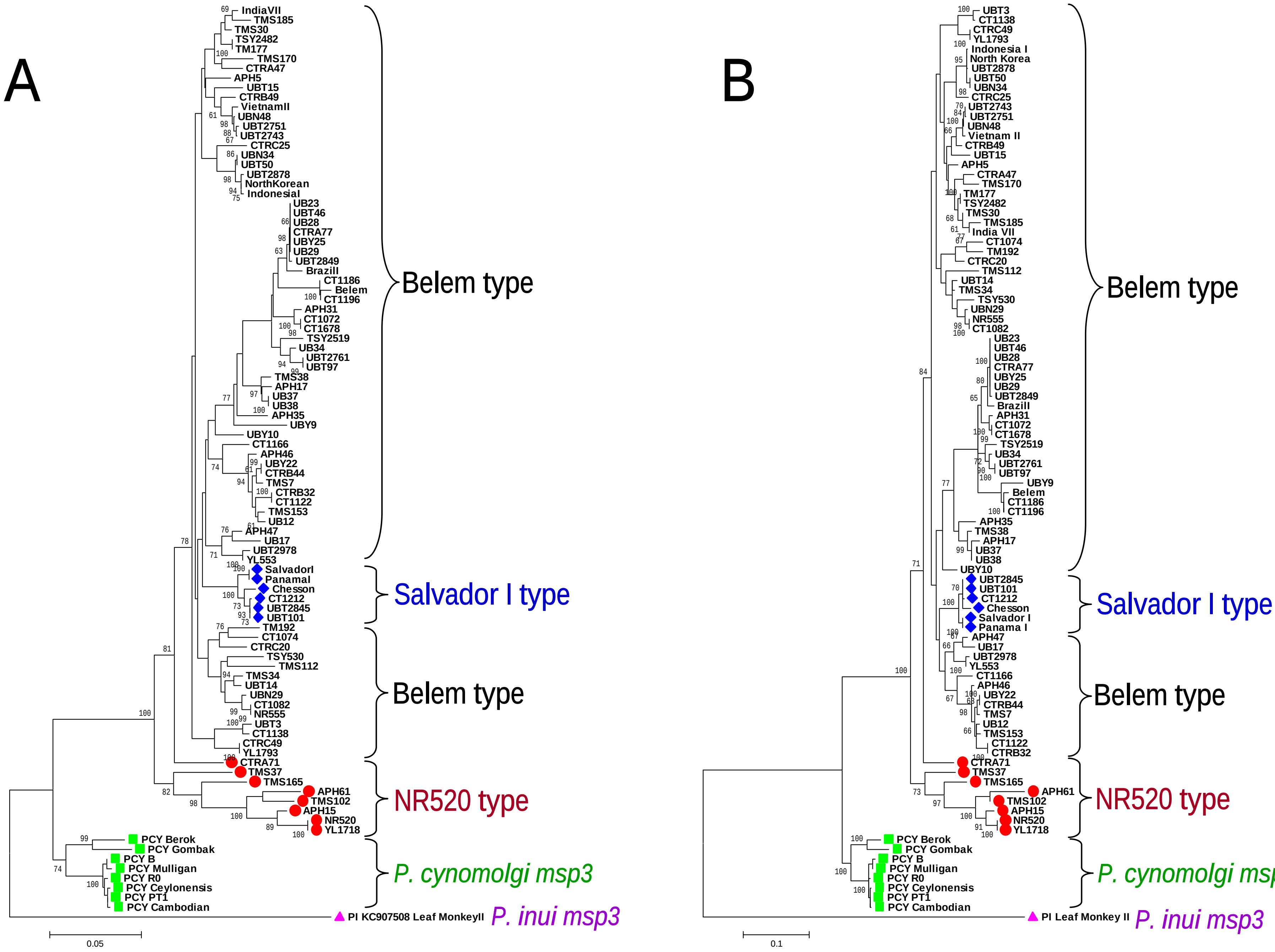

Figure S3. Plots of predicted scores for linear B-cell epitopes across PvMSP3γ.

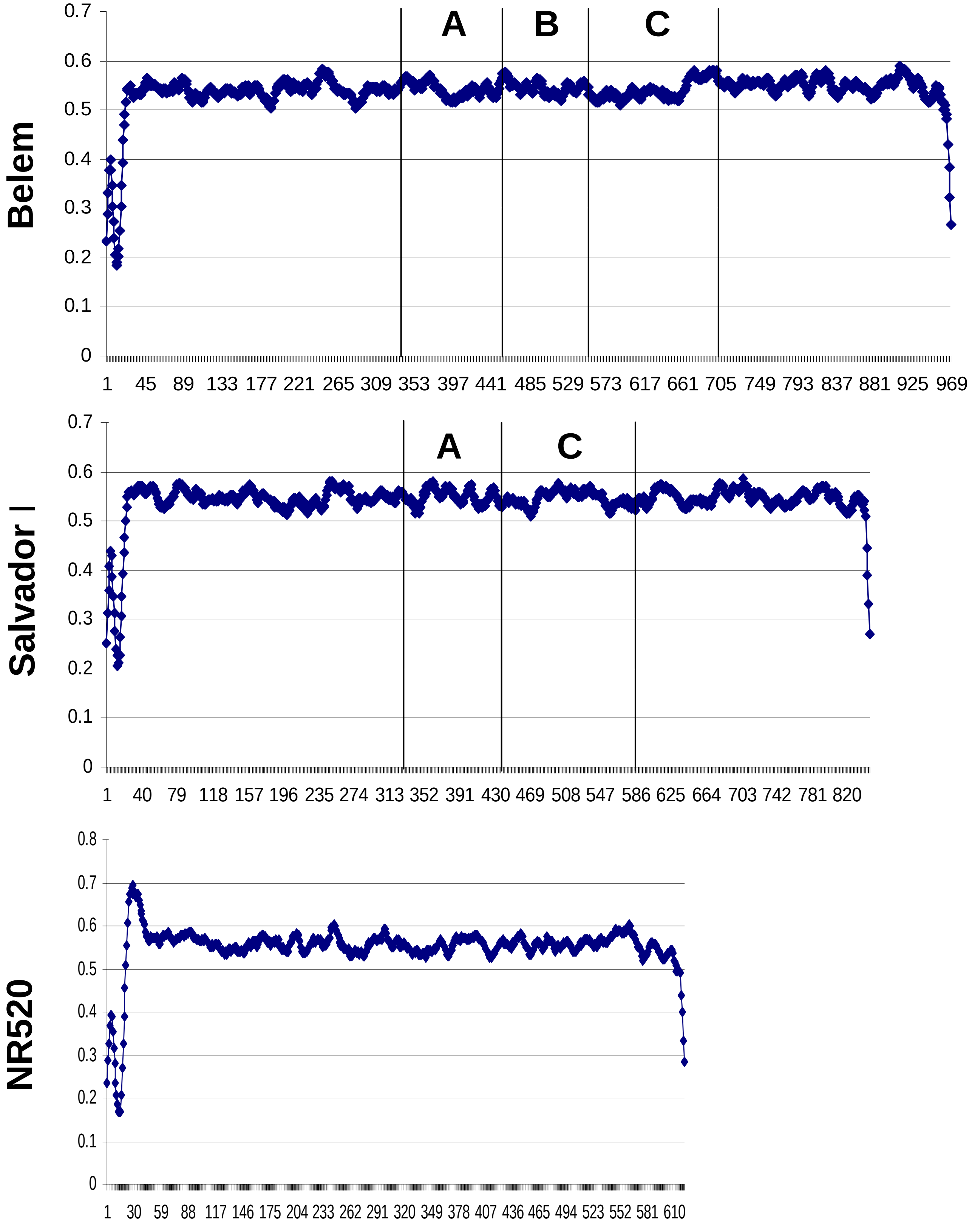

Note: A, B and C are insert domains. Default cutoff value is 0.5.
Numbers in the Y axis are amino acid positions.
